# Tumor-derived alpha-fetoprotein (tAFP) causes immune and metabolic dysfunction in monocyte-derived dendritic cells

**DOI:** 10.1186/2051-1426-3-S2-P210

**Published:** 2015-11-04

**Authors:** Patricia M Santos, Angela Pardee, Greg M Delgoffe, Lisa H Butterfield

**Affiliations:** 1 University of Pittsburgh, Pittsburgh, PA, USA

## Background

Alpha-fetoprotein (AFP) is an oncofetal antigen expressed by over 50% of hepatocellular carcinoma (HCC) tumors. AFP-L3 is the major isoform present in the serum of HCC patients and is associated with poor patient prognosis. While HCC tumor-derived AFP (tAFP) contains >80% of AFP-L3, cord blood serum-derived AFP (nAFP) contains less than 5% of AFP-L3. Previous studies have proposed an immunoregulatory role for AFP on myeloid cells including dendritic cells (DC).

## Methods

Therefore, to test the specific effect of nAFP and tAFP on DC differentiation *in vitro*, peripheral blood monocytes from healthy donors were cultured in the presence of nAFP or tAFP, and DC phenotype and function was assessed after 5 days. We have previously shown that monocytes cultured *in vitro* in the presence of tAFP differentiated into DC that retained a monocyte-like morphology, had decreased expression of surface DC maturation markers, exhibited limited production of inflammatory cytokines, and failed to induce robust T cell proliferative responses. Here, we investigate the mechanisms of tAFP-induced suppressive effects on monocyte-derived DC. Specifically: i) CD1 family surface expression, ii) chemokine production, and iii) DC metabolism.

## Results

Our results show that 5 days after culture, the mRNA and surface expression of CD1a, CD1b, CD1c and CD1d are reduced in nAFP-DC and are further reduced in tAFP-DC. We also show that tAFP-DC had decreased secretion of chemokines CCL1, CCL2, CCL3, CCL4, CCL17, CCL20 and CCL22 in day 6 and/or day 7 supernatants on a per cell basis. Most importantly, we observe reduced mitochondrial mass and a significant defect in mitochondrial oxidative phosphorylation and inhibition of glycolysis in tAFP-DC compared to OVA-DC or nAFP-DC.

## Conclusions

Collectively, these data show profound negative effects of tAFP on DC function. These results help explain some of the immune suppression observed in AFP+ HCC patients and may lead to novel therapeutic approaches to reverse these immunosuppressive effects to improve DC function and enhance anti-HCC immunity.

